# TIM-3 rs1036199 polymorphism increases susceptibility to autoimmune diseases: evidence based on 4200 subjects

**DOI:** 10.1042/BSR20181235

**Published:** 2018-11-23

**Authors:** Rongzeng Liu, Xing Wang, Xiafei Chen, Shengnan Wang, Heqian Zhang

**Affiliations:** 1 Department of Immunology, Medical College, Henan University of Science and Technology, Luoyang 471023, China; 2 Network Information Center, Henan University of Science and Technology, Luoyang 471023, China

**Keywords:** Autoimmune diseases, Meta-analysis, Polymorphism, TIM-3

## Abstract

Conflicting results have been reported regarding differing studies on the association between T-cell immunoglobulin and mucin domain 3 polymorphisms and autoimmune disease. The purpose of the present study was to evaluate the association of TIM-3 rs1036199 (4259 G/T) polymorphism with autoimmune disease susceptibility. A meta-analysis was performed to obtain a more precise evaluation of the association. Ten eligible studies were retrieved by searching PubMed, Embase and Web of Science databases, and statistical analyses were performed using STATA software. The pooled results indicated that TIM-3 rs1036199 polymorphism was significantly associated with an increased risk of overall autoimmune disease in allele comparison (G versus T: OR = 1.59, 95%CI: 1.17–2.17) and heterozygous comparison (GT versus TT: OR = 1.68, 95%CI: 1.37–2.06). Subgroup analyses based on disease type demonstrated that TIM-3 rs1036199 polymorphism was associated with an increased risk of rheumatic arthritis (G versus T: OR = 1.88, 95%CI: 1.45–2.44; GT versus TT: OR = 2.02, 95%CI: 1.53–2.65), especially in Asian populations.

## Introduction

Autoimmune diseases (ADs) are a major health issue worldwide, affecting nearly 10% of the population [1]. Human ADs are often complex diseases caused by the interplay of genetic and environmental factors [2]. It has been widely reported that several different ADs share a significant genetic background [3–5]. A growing body of evidences suggests that T-cell immunoglobulin and mucin domain (TIM) proteins participate in both the regulation of helper T-cell immune response and in ADs [6–12].

TIM-3 is a negative regulator of immune responses and can be expressed on activated Th1 cells, CD8^+^ T cells and at a low level on Th17 cells [13–15]. These cells produce decreased amounts of cytokines or are less proliferative when TIM-3 is activated by galectin-9 [16,17]. Blockade of the TIM-3 signaling pathway restores proliferation and enhances cytokine production in vaccine-induced CD8^+^ T cells [18]. TIM-3 also induces peripheral tolerance through interacting with galectin-9, revealing an inhibitory action on T-cell responses [19,20].

Several studies have demonstrated that TIM-3 polymorphism could be associated with ADs, such as multiple sclerosis (MS), Graves’ disease (GD), Hashimoto’s disease (HD), autoimmune thyroid diseases (AITDs), ankylosing spondylitis (AS), idiopathic thrombocytopenic purpura (ITP), systemic lupus erythematosus (SLE) and rheumatoid arthritis (RA) [21–32]. Results, however, have been inconsistent, possibly due to the low statistical relevance of the individual studies. We, therefore, present a meta-analysis of the published data in order to evaluate the possibility of association between TIM-3 rs1036199 (4259 G/T) polymorphism and AD susceptibility.

## Materials and methods

### Literature search strategy

For the meta-analysis, PubMed, Web of Science and Embase databases were searched without language limitations. The final search was performed on July 7, 2018, using search terms: ‘T cell immunoglobulin and mucin domain 3 OR TIM-3 OR 4259 G/T OR rs1036199’, ‘autoimmune diseases OR autoimmunity’ and ‘polymorphism OR variant’. In order to identify additional eligible studies, references from all relevant articles were also included.

### Inclusion criteria

Selection criteria were independently assessed by two researchers, based on several factors. First, assessment of the association between TIM-3 rs1036199 and ADs; second, only case–control studies were included, and finally that sufficient genotype data were available for odds ratio and confidence interval calculations. Agreement was reached via discussion in the case of any conflicts.

### Data extraction

Data were extracted from the eligible studies by two independent researchers. The following data types were extracted: the first author, publication year, country, ethnicity, disease type, genotyping method, source of control, total number of cases and controls, Hardy–Weinberg equilibrium and amount of cases and controls for every genotype. Any dispute was resolved by discussion.

### Statistical analysis

The Chi-squared test was used to examine the Hardy–Weinberg equilibrium within the control group, with *P*<0.05 considered as a statistically significant disequilibrium. Odds ratio (OR) and 95% confidence interval (CI) were calculated to assess the association between TIM-3 rs1036199 polymorphism and ADs. Pooled OR results were derived from the combination of each study through comparison in allelic (G versus T) and heterozygote (GT versus TT) models. A Z-test was used to determine pooled OR results, and a *P* value <0.05 was considered significant. Heterogeneity was assessed using a standard *Q*-statistic test and an *I*^2^ test was used to quantify inconsistency. If the *P*-value of the *Q*-test was less than 0.1, or an *I*^2^ value >50%, ORs were pooled according to the random-effective model. Otherwise, the fixed-effective model was employed. Sensitivity analyses were conducted toward every genetic model to assess the influence of each individual study on combined ORs by sequentially deleting each study. Additionally, subgroup analysis was stratified by disease, ethnicity and source of control. Publication bias was evaluated by Egger’s test and Begg’s funnel plots. All statistical analyses were performed using STATA software, version 14.0 (StataCorp, College Station, TX).

## Results

### Literature review and description of included studies

**Figure 1:**
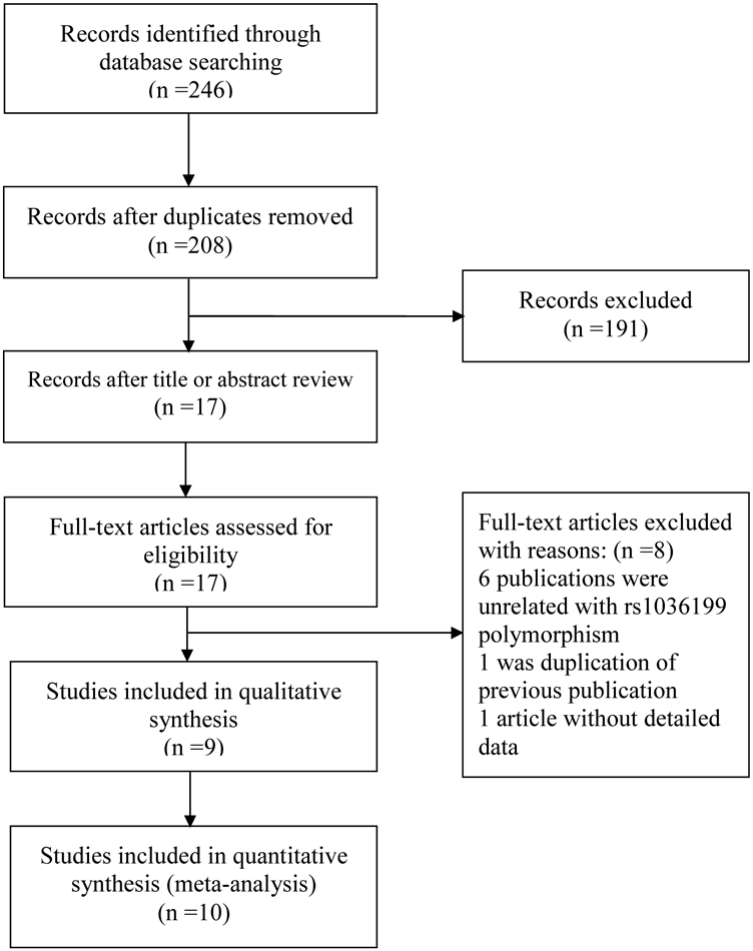
Process of study selection in this meta-analysis

The study selection process is illustrated in Figure 1. In total, 246 relevant studies with the TIM-3 rs1036199 polymorphism and AD were identified through PubMed, Embase and Web of Science. Initially, 38 publications were excluded due to duplication. Next, 191 studies were removed after screening the titles and abstracts. Subsequently, 17 articles were evaluated by reading the full-text and 8 articles were removed because of incomplete or irrelevant data focusing on other TIM-3 polymorphisms [21,25,27,31–35]. Finally, 9 publications meeting the inclusion criteria were selected [22–24,26,28–30,36,37]. In addition, one article included two case–control studies featuring different populations [24,30], so in total, 10 eligible studies, amounting to 2166 ADs and 2034 controls, were enrolled. These investigations presented data on several different autoimmune disease types, such as MS, GD, AITDs, AS, RA and ITP. Among the 10 studies, 9 were conducted in Asian populations and 1 in an African population. The genetic distributions of the control groups in all investigations were consistent with the Hardy–Weinberg equilibrium. The characteristics of the selected investigations are summarized in Table 1.

**Table 1:** Characteristics of the included studies

Study	Year	Country	Ethnicity	Diseases	Genotyping	Source of controls	Case			Control			HWE
							TT	TG	GG	TT	TG	GG	
Pouladian [35]	2017	Iran	Asian	MS	RFLP	HB	102	34	4	116	21	1	0.963
Inoue [22]	2017	Japan	Asian	AITDs	RFLP	HB	273	14	0	66	4	0	0.806
Wang [27]	2014	China	Asian	AS	RFLP	HB	262	20	0	279	19	0	0.570
Liang [24]	2012	China	Asian	GD	PCR-SSP	PB	172	10	0	147	3	0	0.902
Xu [28]	2011	China(Hui)	Asian	RA	PCR-SSP	PB	198	28	0	224	7	0	0.815
Xu [28]	2011	China	Asian	RA	PCR-SSP	PB	90	13	0	103	5	0	0.806
Song [26]	2011	China	Asian	RA	TaqMan	HB	335	31	0	365	24	0	0.530
Radwan [34]	2011	Egypt	African	ITP	RFLP	PB	62	35	0	131	68	9	0.963
Du [21]	2009	China	Asian	ITP	RFLP	HB	178	9	0	120	3	0	0.891
Chas [20]	2004	Korea	Asian	RA	SBE	PB	203	93	0	256	63	0	0.050

Abbreviations: AITD, autoimmune thyroid disease; AS, ankylosing spondylitis; GD, Graves’ disease; HB, hospital-based; HWE, Hardy–Weinberg equilibrium of controls; ITP, idiopathic thrombocytopenic purpura; PB, population-based; PCR-SSP, polymerase chain reaction with sequence specific primers; RA, rheumatoid arthritis; RFLP, restriction fragment length polymorphism.

### Quantitative data synthesis

**Figure 2:**
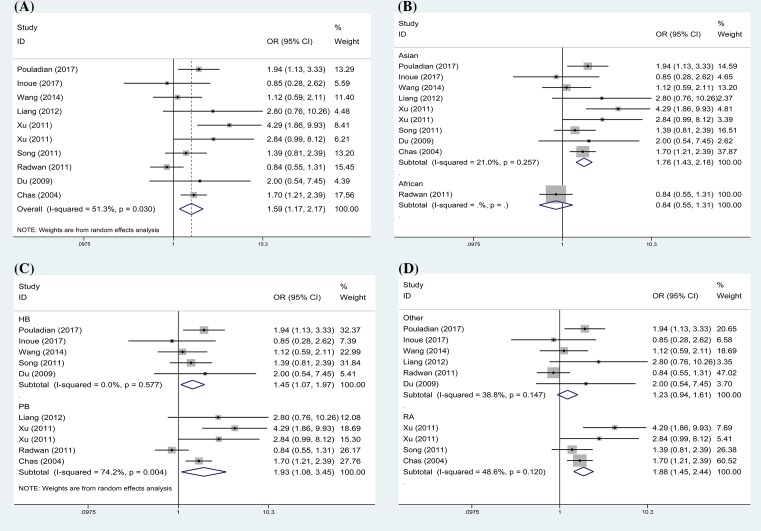
Forest plot of OR with 95% CI for TIM-3 rs1036199 polymorphism and AD risk in allele model (G vs T) (**A**) Overall results; (**B**) stratified analysis by ethnicity; (**C**) subgroup analysis by design of study; (**D**) stratified analysis by AD types.

**Figure 3:**
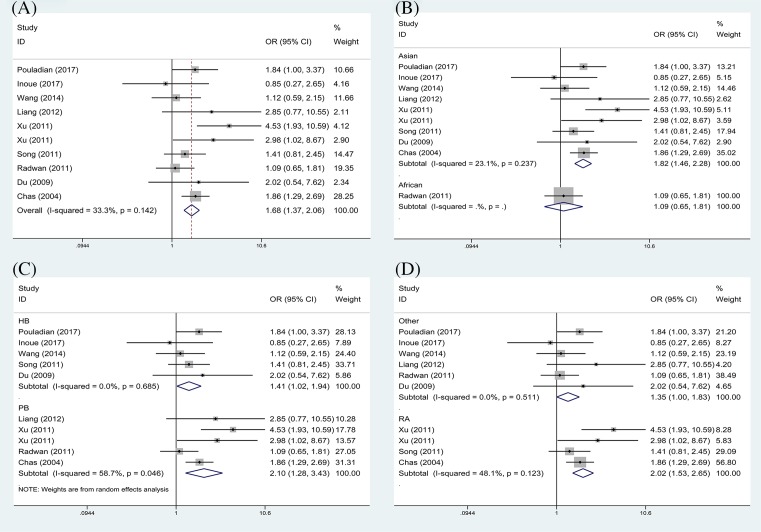
Forest plot of OR with 95% CI for TIM-3 rs1036199 polymorphism and AD risk in heterozygote model (GT vs TT) (**A**) Overall results; (**B**) stratified analysis by ethnicity; (**C**) subgroup analysis by design of study; (**D**) stratified analysis by AD types.

The results are displayed in Table 2. In the overall analysis, TIM-3 rs1036199 polymorphism was associated with an increased risk of ADs in allelic (G versus T: OR = 1.59, 95%CI: 1.17–2.17, Figure 2A) and heterozygous models (GT versus TT: OR = 1.68, 95%CI: 1.37–2.06, Figure 3A). As shown in Table 2, no significant heterogeneity was found in the heterozygous model (*P*=0.142, *I*^2^ = 33.3%), but slight heterogeneity was found in allele model (*P*=0.030, *I*^2^ = 51.3%). Subsequently, subgroup analysis was conducted by ethnicity, source of control and disease type. When subgroup analysis was performed based on ethnicity, significant correlation was detected between rs1036199 polymorphism and increased risk of ADs in Asian populations (G versus T: OR = 1.76, 95%CI: 1.43–2.18; GT versus TT: OR = 1.82, 95%CI: 1.46–2.28, Figures 2B and 3B), but not in African populations. When results were stratified by source of controls, increased risk of AD was detected in both population-based studies (G versus T: OR = 1.93, 95%CI: 1.08–3.45; GT versus TT: OR = 2.10, 95%CI: 1.28–3.43, Figures 2C and 3C) and in hospital-based studies (G versus T: OR = 1.45, 95%CI: 1.07–1.97; GT versus TT: OR = 1.41, 95%CI: 1.02–1.94, Figures 2C and 3C). Moreover, significant associations were reached in the subgroup of rheumatoid arthritis using the two genetic models (G versus T: OR = 1.88, 95%CI: 1.45–2.44; GT versus TT: OR = 2.02, 95%CI: 1.53–2.65, Figures 2D and 3D).

**Table 2:** Summary of OR and 95% CI values for TIM-3 rs1036199 polymorphism and AD risk

		G vs T		GT vs TT	
Variables	*N*	OR (95%CI)	*P/I^2^*(%)	OR (95%CI)	*P/I^2^*(%)
Total	10	**1.59 (1.17–2.17)**	0.030/51.3	**1.68 (1.37–2.06)**	0.142/33.3
**Ethnicity**					
Asian	9	**1.76 (1.43–2.18)**	0.257/21.0	**1.82 (1.46–2.28)**	0.237/23.1
African	1	–	–	**–**	–
**Source of control**					
HB	5	**1.45 (1.07–1.97)**	0.577/0.0	**1.41 (1.02–1.94)**	0.685/0.0
PB	5	**1.93 (1.08–3.45)**	0.004/74.2	**2.10 (1.28–3.43)**	0.046/58.7
**Disease type**					
RA	4	**1.88 (1.45–2.44)**	0.120/48.6	**2.02 (1.53–2.65)**	0.123/48.1
Others	6	1.23 (0.94–1.61)	0.147/38.8	**1.35 (1.00–1.83)**	0.511/0.0

Abbreviations: HB, hospital-based; *N*, number of studies; *P, P* value of *Q*-test for heterogeneity; PB, population-based.

### Sensitivity analysis

**Figure 4:**
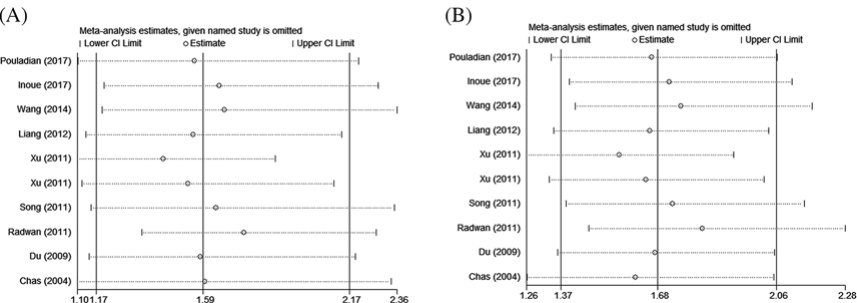
Sensitivity analysis of association between TIM-3 rs1036199 polymorphism and AD risk. (A) allele model; (B) heterozygote model.

Sensitivity analysis was conducted to investigate the individual study’s effect on the pooled ORs. After every individual study was sequentially excluded from the pooled analysis, the results indicated that there was no remarkable change of data for the two models (Figure 4A,B). This provided evidence of the consistency of the results.

### Publication bias

**Figure 5:**
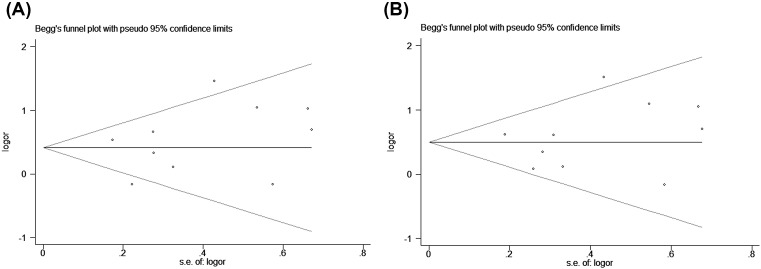
Begg’s funnel plot evaluating evidence of publication bias from the eligible studies. (A) allele model; (**B**) heterozygote model.

Begg’s funnel plots were performed to assess any possible publication bias, and no obvious asymmetry evidence was found according to the shape of the funnel plots (Figure 5A,B). Subsequently, Egger’s linear regression was utilized to quantitatively estimate the publication bias, the *P*-value of Egger’s test indicated a lack of publication bias for rs1036199 polymorphism (*P*=0.374 for the allelic genetic model; *P*=0.510 for the heterozygous genetic model).

## Discussion

TIM-3 gene is located on chromosome 5q33.2 and is mainly expressed in Th1 cells [38]. The TIM-3–galectin-9 signaling pathway induces cell death and ends the Th1 response at tissue sites [39]. Given that TIM-3 can reduce the antigen-specific T-cell responses, we speculate that TIM-3 polymorphism conferred individual risk for ADs by increasing TIM-3 expression and/or enhancing TIM-3 activity.

The 4259 G/T polymorphism is located within exon 3 of TIM-3. A switch from T to G leads to the amino-acid substitution of arginine by leucine [40]. The effects of amino acid substitution at this site remain unclear. Perhaps the amino acid substitution arising from the SNP leads to the alteration of TIM-3 structure and thus influences the immune function of the cell. The variation may also affect the susceptibility to ADs. Most SNPs are associated with more than one autoimmune disease, indicating shared immunological pathways that are disrupted when immune tolerance is broken [3]. However, relatively few studies were conducted on the association between autoimmune diseases and other TIM-3 SNPs. For example, only two publications investigate the relationship between TIM-3 -1541C>T polymorphism and autoimmune diseases [27,30].

To the best of our knowledge, no previous meta-analysis has comprehensively investigated the association between rs1036199 polymorphism and AD risk. Our analysis revealed that TIM-3 rs1036199 polymorphism was significantly associated with an increased overall risk of AD. The AD risk was markedly more pronounced in Asian populations using allelic and heterozygous genetic models. Subgroup analyses based on disease type further revealed that TIM-3 rs1036199 polymorphism was only associated with an increased risk of rheumatoid arthritis and might have no effect on GD or ITP.

There are, however, some limitations to the meta-analysis. First, for several ADs, the sample of studies is small, which may lead to insufficient power to detect a slight association. Second, most of the investigations analyzed were conducted on Asian populations, so further investigation into other ethnic populations is required. In addition, more eligible investigations on different kinds of autoimmune disease are recommended.

In conclusion, our analysis indicates that TIM-3 rs1036199 polymorphism increases the susceptibility to AD in the overall population and in Asian populations. In particular, it shows that the TIM-3 rs1036199 polymorphism is associated with an increased genetic susceptibility to rheumatoid arthritis.

## Abbreviations

95% CI: 95% confidence interval
AITD: autoimmune thyroid disease
AS: ankylosing spondylitis
GD: Graves’ disease
HB: hospital-based
HD: Hashimoto’s disease
HWE: Hardy–Weinberg equilibrium
ITP: idiopathic thrombocytopenic purpura
OR: odds ratio
PB: population-based
PCR-SSP: polymerase chain reaction with sequence specific primers
RA: rheumatoid arthritis
RFLP: restriction fragment length polymorphism
